# New insights into diversity and selectivity of trentepohlialean lichen photobionts from the extratropics

**DOI:** 10.1007/s13199-014-0285-z

**Published:** 2014-06-21

**Authors:** Christina Hametner, Elfriede Stocker-Wörgötter, Martin Grube

**Affiliations:** 1 Department of Organismic Biology, University of Salzburg, Hellbrunnerstraße 34, 5020 Salzburg, Austria; 2 Department of Plant Science, Karl-Franzens-University of Graz, Holteigasse 6, 8010 Graz, Austria

**Keywords:** ITS region, Lichen symbioses, Photobionts, Phylogeny, Temperate regions, Trentepohliaceae

## Abstract

Aerial green algae of Trentepohliaceae can form conspicuous free-living colonies, be parasites of plants or photobionts of lichen-forming ascomycetes. So far, their diversity in temperate regions is still poorly known as it has been mostly studied by phenotypic approaches only. We present new insights in the phylogenetic relationships of lichenized representatives from temperate and Mediterranean parts of Europe by analysis of 18S rRNA and *rbc*L gene fragments, and nuclear ITS sequence data. For this purpose we isolated the trentepohlialean photobionts from lichens representing different genera. Algal cultures from lichenized and free-living Trentepohliaceae were used to design new primers for amplification of the marker loci. We constructed a phylogenetic hypothesis to reveal the phylogenetic placements of lichenized lineages with 18S rRNA and *rbc*L sequences. ITS variation among the clades was substantial and did not allow including them in the general phylogenetic assessment, yet ITS appears to be a promising marker for DNA-barcoding approaches. Specific algae were found in particular lichen but the overall diversity of photobionts was limited. The multilocus tree does not support the current morphological classification of genera in Trentepohliaceae, suggesting that morphology is more variable than previously thought in this group of algae.

**Electronic supplementary material:**

The online version of this article (doi:10.1007/s13199-014-0285-z) contains supplementary material, which is available to authorized users.

## Introduction

The Trentepohliaceae are a widespread family of aero-terrestrial green algae which differs from other green algae in terms of their reproductive structures, phragmoplast-mediated cytokinesis, the lack of pyrenoids in the chloroplast and other characters (Rindi et al. 2009). In particular, the phragmoplasts are otherwise only known from the Charophyceae and from land plants (Chapman et al. 2001). *Trentepohlia* colonies and of their allied genera are frequent on rocks, buildings, tree barks, leaves, stems, and fruits (Printz 1939; Chapman 1984; López-Bautista et al. 2002; Chapman and Waters 2004; López-Bautista et al. 2006; Gaylarde et al. 2006; Nelson 2008). Among the aero-terrestrial algae, the Trentepohliaceae are particularly well-adapted to habitats with high levels of air humidity. These habitats are also frequently colonized by species of lichen-forming fungi which form symbioses with trentepohlialean algae. Nelsen et al. (2011) estimated that approximately 23 % of all lichen-forming fungi are associated with trentepohlialean photobionts. The highest species diversity of Trentepohliaceae is so far reported from tropical to subtropical regions (Thompson and Wujek 1997; Chapman 1984; Chapman and Waters 2004; López-Bautista et al. 2002; Rindi et al. 2010), but it is still unclear how many of these species are also symbionts of lichens.

The free-living representatives of the type genus *Trentepohlia* consists of small shrubby or cushion-like thalli composed of branched filaments. As the cells produce a vast amount of carotenoid pigments in light-exposed locations, the colonies are recognizable from a distance by their orange color. In contrast, lichenized forms of Trentepohliaceae do not develop well-formed filaments as the fungal hyphae control the photobionts, but instead form only short concatenations of cells (Honegger 1998; Chapman and Waters 2004). In lichen thalli, the trentepohlialean photobionts are usually sheltered below an upper fungal layer which protects the algal cells against detrimental UV radiation. Consequently, in the lichenized state, the production of carotenoids is reduced and the green chloroplasts become more distinct. As a result of these alterations, and because sexual stages are usually suppressed, the identification of Trentepohliaceae on the basis of morphological features is difficult.

Molecular analyses have facilitated the classification of lichenized green algae. So far, this has been mostly achieved with coccal photobionts of the family Trebouxiophyceae (e.g. Cordeiro et al. 2005; del Campo et al. 2010; Bock et al. 2011; Ruprecht et al. 2012). Few studies of trentepohlialean photobionts have focused on analysis of the fungal selectivity. So far, partial small subunit rRNA genes and the large subunit of the ribulose-bisphosphate carboxylase gene (*rbc*L) have been used for phylogenetic placement of free-living trentepohlialean algae and in Viridiplantae, especially in the class Ulvophyceae (López-Bautista and Chapman 2003; López-Bautista et al. 2006; Rindi et al. 2009). Moreover, the relationships within the order Trentepohliales and also between the genera *Trentepohlia* and *Printzina* have been analyzed using these two loci. The phylogenetic trees generated with the 18S rRNA gene were poorly resolved at the genus and species level. However, combined analysis with the *rbc*L gene resulted in higher support values for various branches (Rindi et al. 2009). Despite these results, the assignment of named trentepohlialean species to terminal branches remains problematic. These findings were taken as evidence for the insufficiency of the current classification within Trentepohliaceae. Nelsen et al. (2011) discussed a similar situation in their initial molecular study of Trentepohliaceae associated with lichen-forming fungi and used the *rbc*L gene. Their study mainly included tropical lichens and their topology of the phylogenetic hypothesis is not well supported for a majority of the clades. Before it is possible to complete a revision of classification and have a better knowledge of relationships in general, the phylogeny of Trentepohliaceae requires further support with the inclusion of additional markers.

At a low taxonomic level the ITS region of the ribosomal gene cluster has been used in diverse organismal groups. It was therefore our plan to include this locus in studies of Trentepohliaceae. In the context of a larger survey of trentepohlialean lichen symbionts, we were interested in the diversity of trentepohlialean photobionts in lichens from temperate and Mediterranean regions in Europe. Initially, we used published algal-specific-primers and found that the published primers by Nelsen et al. (2011) can amplify both trentepohlialean and trebouxiophycean algae. This represented a problem of a background signal when epithalline algae are abundant in thallus material used for total DNA extractions. Our goal was therefore also to design Trentepohliaceae-specific primers that avoid contamination problems. Using phylogenetic analyses, we then assessed the relationships of trentepohlialean photobionts in selected lichens collected from temperate regions and with free-living representatives.

## Material and methods

### Sampling

Thirty-eight lichens, representing different genera, were collected from various sites in temperate and Mediterranean habitats (Table 1). All voucher specimens, except for *Dimerella pineti* (deposited at the University of Graz, Institute of Plant Science, herbarium GZU 5650), are in the herbarium of E. Stocker-Wörgötter, which is publicly accessible through the Department of Organismic Biology at the University of Salzburg.

**Table 1:** Photobiont strain descriptions of different lichen samples used in this study for phylogenetic analyses

Lichen-taxa	Trentepohliacea-taxa	Location	GenBank accession numbers		
			SSU	ITS	*rbc*L
*Acrocordia gemmata* (156)	*Trentepohlia* sp.	United Kingdom, Scotland, Loch Carron and West Monar, Attadale (N57°23′45.80″ W5°27′1.76″)	JQ618000	JQ617977	JQ617931
*Arthonia cinnabarina* (137)	*Printzina* cf*. lagenifera*	United Kingdom, Scotland, Isle of Skye, Kinloch & Kyleakin (N57°10′55.10″ W 5°48′9.77″)	JQ618002	JQ617961	JQ617944
*Arthonia cinnabarina* (12)	*Printzina lagenifera*	France, Forêt du Cranou (N48°18′57.00″ W 4° 5′42.00″)	-	JQ617952	JQ617932
*Arthonia radiata* (155)	*Trentepohlia* sp.	United Kingdom, Scotland, Loch Carron and West Monar, Attadale (N57°23′45.80″ W5°27′1.76″)	JQ617998	JQ617979	JQ617929
*Arthothelium ruanum* (22)	*Printzina* cf*. lagenifera*	Austria, Salzburg, Bluntautal (N47°33′59.76″ E13° 6′00.52″)	JQ618017	JQ617960	JQ617914
*Cystocoleus ebeneus* (1)	*Trentepohlia* sp.	Austria, Salzburg, Krimmler waterfalls (N 47°12′41.25″ E12°10′6.13″)	JQ617982	JQ617945	JQ617917
*Cystocoleus ebeneus* (2)	*Trentepohlia* sp.	Austria, Salzburg, Krimmler waterfalls (N 47°12′41.25″ E12°10′6.13″)	JQ617983	JQ617946	-
*Dimerella pineti* (65)	*Printzina* sp.	Austria, Styria, Grazer uplands, Dürrgraben (N47°07′15″ E015°28′05″)	JQ618011	JQ617950	-
*Graphis scripta* (13)	*Printzina lagenifera*	Austria, Salzburg, Bluntautal (N47°33′59.76″ E13°6′00.52″)	JF727812	JF727811	JF727813
*Graphis scripta* (14)	*Printzina lagenifera*	Italy, Friaul, Sella Nevea Pass after pass altitude (N46°23′31.13″ E13°28′29.21″)	JF727815	JF727814	JF727816
*Graphis scripta* (19)	*Printzina lagenifera*	Austria, Salzburg, Bluntautal (N47°33′59.76″ E13°6′00.52″)	JQ618004	JQ617959	JQ617940
*Graphis scripta* (20)	*Printzina lagenifera*	Austria, Salzburg, Bluntautal (N47°33′59.76″ E13°6′00.52″)	JQ618005	JQ617956	JQ617938
*Graphis scripta* (21)	*Printzina lagenifera*	Austria, Salzburg, Bluntautal (N47°33′59.76″ E13°6′00.52″)	JQ618008	JQ617958	JQ617943
*Graphis scripta* (27)	*Printzina lagenifera*	Austria, Salzburg, Bluntautal (N47°33′59.76″ E13°6′00.52″)	JQ618007	JQ617957	JQ617939
*Graphis scripta* (28)	*Printzina lagenifera*	Austria, Salzburg, Bluntautal (N47°33′59.76″ E13°6′00.52″)	JQ618012	JQ617951	JQ617915
*Graphis scripta* (29)	*Printzina lagenifera*	Austria, Salzburg, Bluntautal (N47°33′59.76″ E13°6′00.52″)	JQ618009	JQ617953	JQ617937
*Graphis scripta* (30)	*Printzina lagenifera*	Austria, Salzburg, Bluntautal (N47°33′59.76″ E13°6′00.52″)	JQ618006	JQ617955	JQ617941
*Graphis scripta* (143)	*Printzina lagenifera*	United Kingdom, Scotland, Isle of Skye, Kinloch & Kyleakin (N57°10′55.10″ W5°48′9.77″)	JQ618003	JQ617954	JQ617942
*Gyalecta jenensis* (25)	*Trentepohlia aurea*	Austria, Salzburg, Paß Lueg near Golling (N47°34′50.23″ E13°11′6.43″)	JQ618016	JQ617948	JQ617916
*Mycoporum sparsellum (*136)	*Trentepohlia* sp.	United Kingdom, Scotland, Isle of Skye, Kinloch & Kyleakin (N57°10′55.10″ W5°48′9.77′)	JQ617997	JQ617978	JQ617930
*Opegrapha atra* (149)	*Trentepohlia* sp.	United Kingdom, Scotland, Kyle of Lochalsh, Duirinish (N 57°19′9.00″ W5°40′13.06″)	JQ618014	JQ617963	JQ617933
*Opegrapha atra* (151)	*Trentepohlia* sp.	United Kingdom, Scotland, Kyle of Lochalsh, Duirinish (N 57°19′9.00″ W5°40′13.06″)	JQ618013	JQ617947	JQ617934
*Pyrenula laevigata* (26)	*Printzina* sp.	Austria, St. Bartholomä, Königssee (N47°32′45.46″ E12°58′11.61″)	JQ618010	JQ617949	JQ617913
*Pyrenula laevigata* (135)	*Printzina* sp.	United Kingdom, Scotland, Isle of Skye, Kinloch & Kyleakin (N57°10′55.10″ W5°48′9.77″)	JQ618015	JQ617962	JQ617935
*Roccella decipiens* (7)	*Trentepohlia* sp.	Spain, Gran Canaria, Jardin Botánico Canario Viera y Clavijo (N28°3′55.73″ W15°27′36.60″)	JQ617992	JQ617967	JQ617920
*Roccella galapagoensis* (8)	*Trentepohlia* sp.	Spain, Gran Canaria, Jardin Botánico Canario Viera y Clavijo (N28°3′55.73″ W5°27′36.60″)	JQ617991	JQ617966	JQ617921
*Roccella linearis* (5)	*Trentepohlia* sp.	Spain, Gran Canaria, Andés Verdes (N27°50′20.11″ W15°33′45.77″)	JQ617987	JQ617968	JQ617924
*Roccella lirellina* (10)	*Trentepohlia* sp.	Spain, Gran Canaria, Jardin Botánico Canario Viera y Clavijo (N28°3′55.73″ W15°27′36.60″)	JQ617984	JQ617964	JQ617918
*Roccella maderensis* (5)	*Trentepohlia* sp.	Portugal, Azoren, Fortress of São João Baptista (N38°39′5.19″ W27°13′36.71″)	JQ617996	JQ617971	JQ617925
*Roccella* cf. *montagnei* (9)	*Trentepohlia* sp.	Spain, Gran Canaria, Jardin Botánico Canario Viera y Clavijo (N28°3′55.73″ W15°27′36.60″)	JQ617994	JQ617976	JQ617923
*Roccella phycopsis* (7)	*Trentepohlia* sp.	Malta, Ggantija Tempel Gozo (N36°2′50.08″ E14°16′8.77″)	JQ617988	JQ617975	-
*Roccella phycopsis* (6)	*Trentepohlia* sp.	Spain, Gran Canaria, Andés Verdes (N27°50′20.11″ W15°33′45.77″)	JQ617986	JQ617969	JQ617922
*Roccella tinctoria* (2)	*Trentepohlia* sp.	Spain, Teneriffa, Puertito de los Silos (N28°22′29.19″ W16°48′30.98″)	JQ617993	JQ617972	JQ617927
*Roccella tinctoria* (4)	*Trentepohlia* sp.	Spain, Teneriffa, Puertito de los Silos (N28°22′29.19″ W16°48′30.98″)	JQ617985	JQ617970	-
*Roccella tinctoria* (6)	*Trentepohlia* sp.	Portugal, Azoren, São Sebastião (N38°39′46.53″ W27° 5′35.97″)	JQ617989	JQ617974	JQ617926
*Roccella tinctoria* (11)	*Trentepohlia* sp.	Spain, Gran Canaria, Jardin Botánico Canario Viera y Clavijo (N28°3′55.73″ W15°27′36.60″)	JQ617990	JQ617965	JQ617919
*Roccella tuberculata* (8)	*Trentepohlia* sp.	Portugal, Azoren, Fortress of São João Baptista (N38°39′5.19″ W27°13′36.71″)	JQ617995	JQ617973	-
*Thelotrema lepadinum* (144)	*Trentepohlia* sp.	United Kingdom, Scotland, Isle of Skye, Kinloch & Kyleakin (N57°10′55.10″ W5°48′9.77″)	JQ617999	JQ617980	JQ617928

### Culture experiments

Trentepohlialean photobionts of selected lichens (see Online Resource 1) were isolated according to the “Yamamoto-method” (Yamamoto 1990) with the modifications specified in Stocker-Wörgötter (2002). Fragments of lichen thalli (of 2–3 mm size) were washed in sterile bi-distilled water for 15 min, then one drop of Tween 80 was added to the water and the fragments were washed again for 10 min. After the transfer to fresh bi-distilled water, the fragments were washed for another 20 min by agitating in water using a magnetic stirrer. The fragments were then gently homogenized in several drops of sterile water with an autoclaved mortar and pestle. This suspension was filtered first through a sieve with 500 μm mesh-size and then through a sieve with 150 μm mesh-size. Individual lichen pieces (around 150 μm in size) were picked by a sterile bamboo stick and transferred to test tubes with slanted solid nutrient media under a stereomicroscope.

The isolates were grown on three different nutrient media for 5–6 months under a changing light dark regime of 14 h at 20 °C and 10 h at 15 C° with a light intensity of 100 μE m^−2^ s^−1^. We used modified Bold’s Basal Medium (BBM; Deason and Bold 1960), Woods Hole MBL (WHM; Nichols 1973), and optimal *Haematococcus* medium (OHM; Fábregas et al. 2000). After the Trentepohliaceae-colonies reached a size of approximately 5 mm in diameter, some algal filaments were removed with a sterile inoculation needle for subculture in sterilized 50 ml-Erlenmeyer flasks with liquid modified BBM, WHM or OHM, and plugged with sterile cotton. Flask necks were covered with aluminium foil to avoid contaminations. The isolates were further incubated for several months in a culture chamber under the same conditions as mentioned and specified above.

### Molecular methods

Trentepohlialean filaments of the subcultures were transferred to Eppendorf tubes using a sterile inoculation needle in a clean bench and afterwards dried using the speed-vac UNIVAPO 150 ECH (UniEquip, Planegg, Germany). The dried filaments were frozen with liquid nitrogen to facilitate the grinding procedure with the grinding mill MM301 (Retsch GmbH, Haan, Germany). DNA was extracted using a modified CTAB-method (Doyle and Doyle 1987). The modifications included the addition of 400 μl CTAB buffer, which contained 1 % Polyvinylpyrrolidone (PVP-40; Sigma Aldrich, Vienna, Austria) instead of 2-mercaptoethanol, and 2 μl RNase A (10 mg/ml; Fermentas, Vienna, Austria) directly onto the grinded material. Additionally, the samples were centrifuged at 13,300 rpm for 5 min after the preheating procedure and the chloroform isoamyl alcohol (CI) extraction, the CI extraction was carried out twice and step 7 of the original CTAB-method was omitted. Furthermore, the pellet was washed twice with 500 μl 70 % ethanol at 12,000 rpm for 2 min, afterwards dried at 50 °C for 5 min and dissolved in appropriate volume of double-distilled water.

The PCR was initially accomplished with universal primers and parameters as used in Rindi et al. (2009) for the 18S rRNA and *rbc*L genes. For amplification of the ITS region, we initially used the primers ITS5 (White et al. 1990) and ITS4 (White et al. 1990). PCR-products were cleaned with QIAquick PCR product purification Kit (Qiagen, Hilden, Germany) and sequenced by Macrogen Inc. (Korea). The obtained sequences were classified using NCBI Blast searches, and then aligned with sequences retrieved from the NCBI GenBank, representing both diverse green algal groups (e.g. Chlorophyceae, Trebouxiophyceae, Trentepohliaceae, etc.) and lichenized fungi (e.g. Arthoniaceae, Graphidaceae, etc.) with Geneious (Drummond et al. 2011). After the inspection by eye, the alignments of each of the three markers (18S rRNA and *rbc*L genes, nuclear ITS) were used to find appropriate regions for the design of Trentepohliaceae-specific primers.

To apply and test the newly designed primers, total DNA was extracted from thallus fragments by the modified CTAB-method as mentioned above, and using the Plant Mini DNA-isolation Kit (Qiagen). In addition, DNA was also extracted of four samples of cultured, free-living Trentepohliaceae from the culture collection of algae in Göttingen, Germany (strains SAG 73.90, SAG 25.83, SAG 20.94 and SAG 483–1), of the cultured free-living *Trentepohlia iolithus* strain ASIB505 obtained from the culture collection of Prof. Dr. Gärtner (Innsbruck, Austria) and single colonies of free-living *Printzina* cf. *lagenifera* collected from Almbachklamm, Salzburg, Austria (Acc.no. JX675739). The amplification of the 18S rRNA gene was performed with various combinations of primers at an optimal annealing temperature of 57 °C (see Table 2). Nested PCR was performed occasionally, when starting material was scant. The ITS region were amplified with similar conditions as used for the 18S-amplification, but with an annealing temperature at 56 °C.

**Table 2:** Primer information containing primer name, amplified gene region, primer sequence, source of primers and PCR-product size

Combination	Primer name	Gene	Primer sequence	Source of primers	Product size (bp)
1	Tre18S_N1_for	18S rRNA	5′CCC GAC CTT CGG TGA ATC3′	this lab	~ 820
	CHtrente1.rev	18S rRNA	3′CCA CCT CCG ATC CCT AGT5′	this lab	
2 (nested)	Tre18S_N2_for	18S rRNA	5′TAG GGT AGT GGC CTA CCG3′	this lab	~ 700
	CHtrente0.rev	18S rRNA	3′GTC GAG ACT ACG ACG GT5′	this lab	
3	Tre18S_N5a_for	18S rRNA	5′TAG CAT GGG ATG ACA CGA TAG GA3′	this lab	~ 760
	CHtrente2.rev	18S rRNA	3′ACA AAG CTC TAG CCC CAT CA5′	this lab	
4 (nested)	Tre18S_N5_for	18S rRNA	5′GGA TGA CAC GAT AGG ACT TCG3′	this lab	~ 750
	CHtrente2.rev	18S rRNA	3′ACA AAG CTC TAG CCC CAT CA5′	this lab	
5	CHtrente1.for	ITS	5′ACT AGG GAT CGG AGG TGG3′	this lab	~ 700
	ITS4	ITS	3′TCC TCC GCT TAT TGA TAT GC5′	White et al. (1990)	
6	CHtrente2.for	ITS	5′TGA TGG GGC TAG AGC TTT GT3′	this lab	~ 900
	ITS4	ITS	3′TCC TCC GCT TAT TGA TAT GC5′	White et al. (1990)	

For *rbc*L-amplification, PCR was performed first with TrerbcL_mos_for (5′ GAA GCW ATT CCR GGA GAA G 3′) and TrerbcL_mos_rev (3′ CAT CCA TTC TTG AGW AAA GAA TAC 5′), and for semi-nested PCR with the internal reverse primer TrerbcL_sel_rev (3′ GAT AGT CGT GCA TRA YAA TTG G 5′); all cycles using a 50 °C annealing temperature.

### Sequence alignment and phylogenetic analyses

The DNA sequences of the lichenized Trentepohliaceae and the free-living trentepohlialean algae were aligned using the program Geneious. We used two representatives of the order Ulvales as outgroup of the 18S rRNA and *rbc*L genes. Nucleotide sequences of all samples used in this study have been submitted to NCBI GenBank (Accession numbers of the photobionts see Table 1).

The alignments in Fasta-format were converted into Nexus-files by the web-portal Alter alignment (Glez-Peña et al. 2010). Each dataset were analyzed with the program jModelTest 2.1.1 using Akaike Information Criterion (AIC) scores (Darriba et al. 2012; Guindon and Gascuel 2003) to find GTR + G + I as the optimal substitution model for the phylogenetic analyses. The analyses of each single dataset were performed by Cipres Science Gateway (Miller et al. 2010) with MrBayes v.3.1.2. (Huelsenbeck and Ronquist 2001; Ronquist and Huelsenbeck 2003). Analyses of individual ITS-datasets for clades R with Ld1, clade Gs, clade GPD, clades Ta with Td, and clades Ce with Ld2, employed different substitution models and settings, which are described in Online Resource 1. For the analysis of the *rbc*L-sequence alignment, parameters of the GTR + G + I model were adjusted for each codon position. For each of the analyses, 3 000 000 generations were generated, every 1000th tree was sampled and the initial 750 trees were discarded as burn-in. The combined analysis of the marker regions 18S rRNA and *rbc*L contained the same 52 sequences (see Online Resource 2) as used for the separate analyses, and was computed in BEAST 1.7.4 (Drummond and Rambaut 2007) with the appropriate settings of each locus specified for the separate calculations. Three parallel analyses were run for 6 000 000 chains, which were performed under a strict clock, and every 1000th tree was sampled. The three obtained log-files were controlled with the program Tracer v1.5 (Rambaut and Drummond 2007). The tree-files with branch lengths in units of substitutions of each run were combined with a burn-in of 1200 to one common tree-file using LogCombiner1.7.4 in BEAST. Finally, this file was used to summarize the sampled trees to a maximum clade credibility tree with the setting to reflect the posterior median node heights for the clades by TreeAnnotator1.7.4 in BEAST.

The datasets were also analyzed by maximum parsimony with PAUP* (Swofford 2003). The combined Paup-analysis of 18S rRNA and *rbc*L genes was computed under heuristic search, 100 random addition sequence replicates with one trees held at each step, tree bisection reconnection (TBR) and no more than five trees per replicate (treescore ≥1) were saved. Bootstrapping (Felsenstein 1985) was performed with 1,000 replicates. All trees were illustrated using the program FigTree v1.3.1 (Rambaut 2006–2009).

## Results

By using several loci we wanted to increase the phylogenetic resolution of Trentepohliales. So far, this has been achieved with concatenated 18S rRNA and *rbc*L sequences, which increased the support of clades which did not resolve well in separate analyses of individual loci (see Online Resource 3). It was not possible to include the newly generated ITS sequences in the analysis of the whole dataset due to the variation being too high for reliable alignments and also the sample size differed between the data sets. We therefore decided to use ITS to check for congruence with the 18S-*rbc*L phylogeny and for the resolution of terminal branches (see below).

**Fig. 1:**
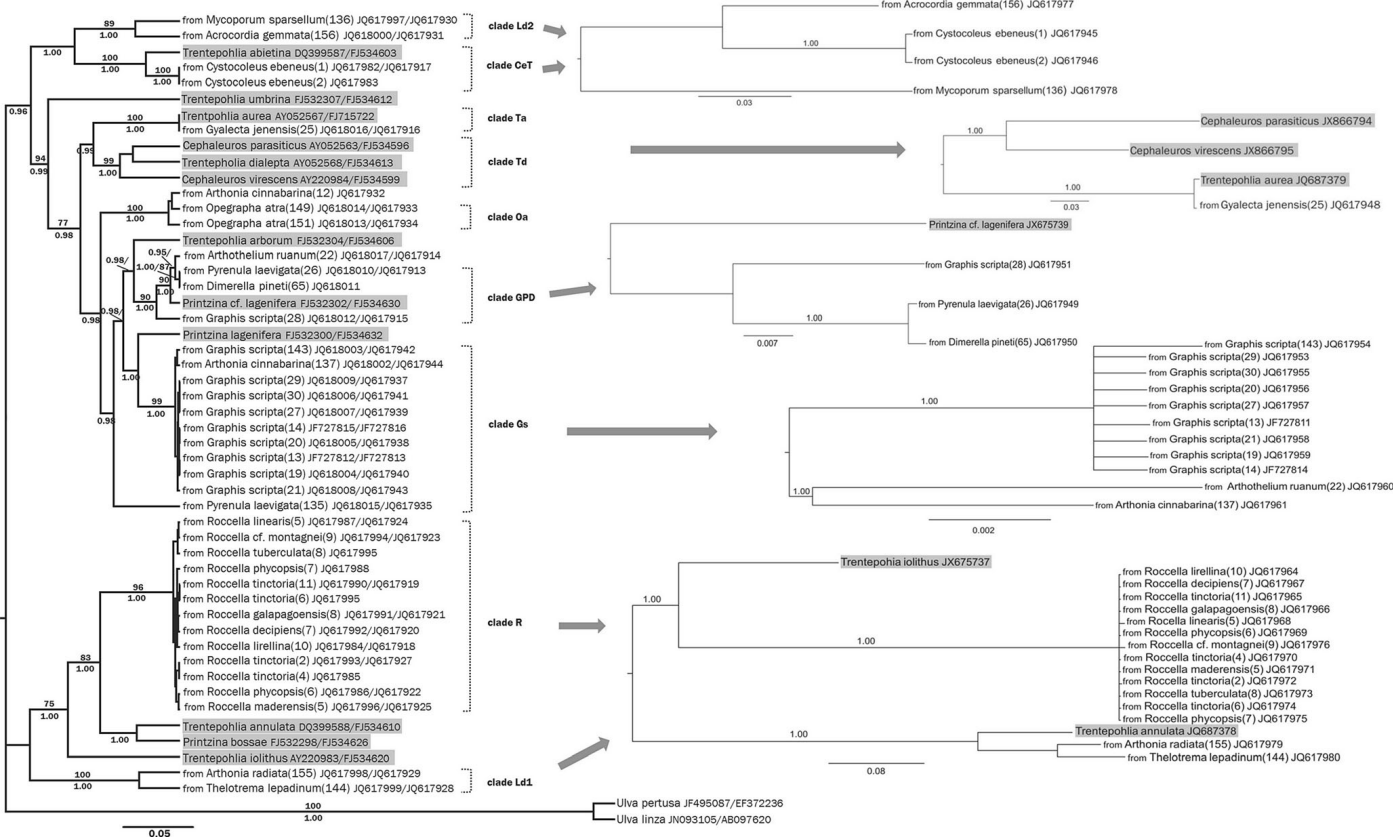
Mid-point rooted maximum clade credibility tree of lichenized and free-living Trentepohliaceae calculated with the concatenated datasets of marker regions 18S rRNA and *rbc*L using Bayesian MCMC analysis implemented in BEAST. Branches with bootstrap support (MPB) ≥70 % and posterior probabilities (PP) ≥0.95, which reflect the posterior median node heights for the clades, were considered as strongly supported. MPB values were illustrated above the branches, whereas PP values were shown below. The tree was rooted using two outgroup sequences of the genus *Ulva*. Single consensus trees with posterior probability values (PP) ≥0.95 of different clades using the ITS rDNA are shown on the right side of the combined tree. The bars specify the substitutions per site. The trentepohlialean algae in free-living stage are grey-shaded and were used as reference for the identification of the photobiont strains

The 18S-*rbc*L phylogeny (Fig. 1) reveals several major clades with high support values (MPB 96 %/1.00 PP and MPB 99 %/1.00 PP). Clade R is closely related to strains representing *Printzina bossae*, *Trentepohlia annulata* (1.00 PP), and *T. iolithus* (MPB 75 %/1.00 PP). The sister clade to this group is clade Ld1 (MPB 100 %/1.00), which is not fully supported by the separate analysis of the ITS-dataset, according to which *T. annulata* groups together with clade Ld1. An incongruence between the overall and ITS- phylogeny can also be found in clade Gs. The photobiont of *Pyrenula laevigata* (135) JQ617962 is not included in the *Graphis scripta*-group of the ITS-tree calculated with the alignment of all ITS-sequences (data not presented); in contrast, this strain shows a close relationship to the trentepohlialean alga lichenized with Arthothelium ruanum (22) JQ617960. The free-living *Printzina lagenifera* forms a sister species to the monophyletic clade Gs, on the basis of the combined analysis. Clade GPD is composed of the photobionts from *Dimerella pineti* (65), *Graphis scripta* (28), *Pyrenula laevigata* (26), and the free-living alga *P.* cf. *lagenifera* (MPB 90 %/1.00) and is closely related to the photobiont of *Arthothelium ruanum* (22), the free-living *Trentepohlia arborum* and clade Gs. The remaining clades (OaA, Td, Ta, CeT and Ld2) of the maximum clade credibility tree consist of the same lichenized algal clusters, as shown in the Bayesian analysis using the *rbc*L-dataset. Clade Td represents a close relationship to Ta with a high posterior probability of 0.99, as well as clade CeT to clade Ld2 (1.00 PP). Despite the support of the CeT-Ld2 relation, the separate ITS-consensus tree reveals only a high supported relation within the algal strains of *Cystocoleus ebeneus* No.1 and 2 (1.00 PP), and uncertain associations from the photobionts of *Mycoporum sparsellum* (136) and *Acrocordia gemmata* (156). *Trentepohlia umbrina* (free-living) forms an individual branch which agrees with the 18S-*rbc*L-analysis.

## Discussion

Previous phylogenetic analyses of trentepohlialean algae revealed their position to be in the Ulvophyceae in Chlorophyta (López-Bautista and Chapman 2003; Leliaert et al. 2012). However, more detailed molecular analyses within the order Trentepohliales showed that the taxonomy was not clearly settled, especially in the case of the genera *Printzina* and *Trentepohlia* (Rindi et al. 2009)*.* As expected, our initial separate, single-locus analyses of lichenized and free-living Trentepohliaceae were poorly supported at internal branches, and did not resolve relationships within the family. The 18S-*rbc*L tree received higher support, which agrees with the results of Rindi et al. (2009) for free-living Trentepohliaceae. They recommended the addition of another molecular dataset, such as ITS or D1-D2 28S rRNA, for a better resolution within some problematic taxa, including *Printzina lagenifera* and *Trentepohlia arborum* (Rindi et al. 2009). Therefore, it appeared reasonable to include ITS-sequences in the analyses because this locus was informative at a lower taxonomic level in other green algal orders (Coleman and Mai 1997; Blaha et al. 2006; Van Der Strate et al. 2008). ITS, in particular the ITS2 region, was also considered to be promising for the DNA-barcoding of green algae, besides the suggested chloroplast genes *rbc*L and *tuf*A, the LSU of rDNA and the mitochondrial COI gene (Leliaert et al. 2009; Grube and Muggia 2010; Hall et al. 2010; Saunders and Kucera 2010; Škaloud and Peksa 2010; Fucikova et al. 2011; Saunders and McDevit 2012). Our analyses actually support the use of ITS for barcoding in Trentepohliaceae. Variation between species appears to be rather high while there is limited variation within the branches we consider as species. In lichens, DNA barcoding approaches could be promising for an assessment of algal selectivity and uniformity of photobionts in individual lichen thalli.

All phylogenetic analyses in this study showed that *Roccella* species (clade R) only associate with a single and distinct trentepohlialean species. The selectivity of this species might correlate with the coastal habitat of the genus *Roccella*. The collection sites comprise various islands which were distantly located about 3,700 km from one another. The roccelloid lichens so far studied contain an undescribed trentepohlialean species as photobiont, which has not yet been found in association with other lichenized ascomycetes. A more detailed determination of this photobiont was not possible without morphological examination of cultured material. It remains to be shown if this algal species is also characterized by a higher salt tolerance compared with the other cultured Trentepohliaceae. *Printzina bossae*, *Trentepohlia annulata*, and *T. iolithus* are sister species of the algal partner from the studied *Roccella* species, but apparently not conspecific with it. The phylogenetically related strains of clade R- photobionts prefer completely different habitats to *Roccella*. Thus, *Printzina bossae*, was collected from the bark of unidentified trees located on an island of Barro Colorado and from a public park in Gamboa (Panama) (Rindi et al. 2008). Other specimens were collected from the bark of an oil palm (French Guiana), bark of *Cryptomeria* sp. (Azores), as well as the bark of an unidentified tree in Florida (U.S.A) (Rindi and López-Bautista 2008; Nelsen et al. 2011).

The free-living alga *Trentepohlia annulata*, also related to clade R, has been reported from vastly distant localities such as Trebitsch (Central Europe) and French Guiana (South America), although the affiliation to *T. annulata* of the specimen from French Guiana has not been 100 % confirmed (Rindi et al. 2009). The *T. annulata*, found in Trebitsch, was growing on the roots and on a cross-section of a conifer trunk (Prat 1914). This algal species was also identified in the apothecia and as an epiphyte on the lichen *Micarea misella* (Voytsekhovich et al. 2011), which normally associates with coccoid algal photobionts. Finally, the cultured strain *T. annulata* No. 20.94 of the algal collection SAG originated from Czechoslovakia.

The cosmopolitan *Trentepohlia iolithus*, also related to clade R, forms red coatings on rocky cliffs of the British Isles, tree bark, periodically submerged rocks along calcareous streams (John 2002) and also grow on limestone used to face buildings, old concrete and cement walls in unpolluted urban areas (Rindi and Guiry 2002; Rindi et al. 2003). The algal symbiotic partners of *Arthonia radiata* and *Thelotrema lepadinum* (clade Ld1) were specified as unknown *Trentepohlia*-species based on relationships with three free-living Trentepohliaceae grouped in clade R and with the NCBI Blast results.

The maximum clade credibility tree computed in BEAST clearly revealed (support values of MPB 100 % and PP 1.00) that the lichenized fungus *Cystocoleus ebeneus* associates with trentepohlialean representatives closely related to *Trentepohlia abietina* (clade CeT)*.* Free-living *T. abietina* has so far been reported from temperate and tropical regions throughout the world (Wildeman 1900; Jose and Chowdary 1980; Tracanna 1989; Ettl and Gärtner 1995; John 2002; Rindi et al. 2005; Rindi et al. 2006). In the free-living stage, this species is restricted to bark as a substrate where it forms distinct yellow-orange patches (Rindi et al. 2005; Rindi et al. 2008). The photobionts of *Acrocordia gemmata* and *Mycoporum sparsellum* (clade Ld2) formed a sister clade to the *Trentepohlia abietina-*strains and are as yet unnamed trentepohlialean lineages.

According to sequence data, the photobiont strains associated with ascomycetes of the lichen species *Graphis scripta* (clade Gs) were identified as *Printzina lagenifera*, and this is confirmed based on morphological observations of cultured strains (Hametner et al. 2014). This mycobiont does not seem to be specific for a single trentepohlialean clade and has the ability to switch photobionts. For example, specimen *Graphis scripta* (28), which was collected at the same locality in Austria as the other specimens from Austria, was found to associate with *Printzina* cf. *lagenifera*. This alga is also found as the photobiont of *Arthothelium ruanum, Dimerella pineti* and *Pyrenula laevigata* (clade GPD). Nelsen et al. (2011) also discovered two specimens of the genus *Graphis* in symbiosis with the alga *P.* cf. *lagenifera*.

The lichen *Arthonia cinnabarina* is another example of a species that can switch algae. It can associate with the algal *Printzina lagenifera* (clade Gs), as well as another lineage of the genus *Printzina* which was found in *Arthonia cinnabarina* (12), and which we assigned to this genus on the basis of the NCBI Blast searches and concatenated phylogenetic analyses with all three loci (data not presented). Based on the 18S-*rbc*L analysis of this study, the photobiont of *A. cinnabarina* (12) clustered together with algal strains of the lichen *Opegrapha atra* which were determined as *Trentepohlia* sp. (clade Oa). This grouping appeared due to the fact that only the *rbc*L-sequence of the *Arthonia*-*cinnabarina*- photobiont was involved as a substantial factor in the combined 18S-*rbc*L analysis.

The free-living *P. lagenifera* seems not to be very specific for substrata (e.g. occurring on the barks of trees, cement, limestone, etc.) and has a very wide distribution in both temperate and tropical regions (Rindi and Guiry 2002; Rindi et al. 2006; Rindi et al. 2008). All lichen species in our study were collected in Europe, which led to the conclusion that in this region *Printzina lagenifera* is a common algal species and tends to be an adequate photobiont for several lichens.

**Fig. 2:**
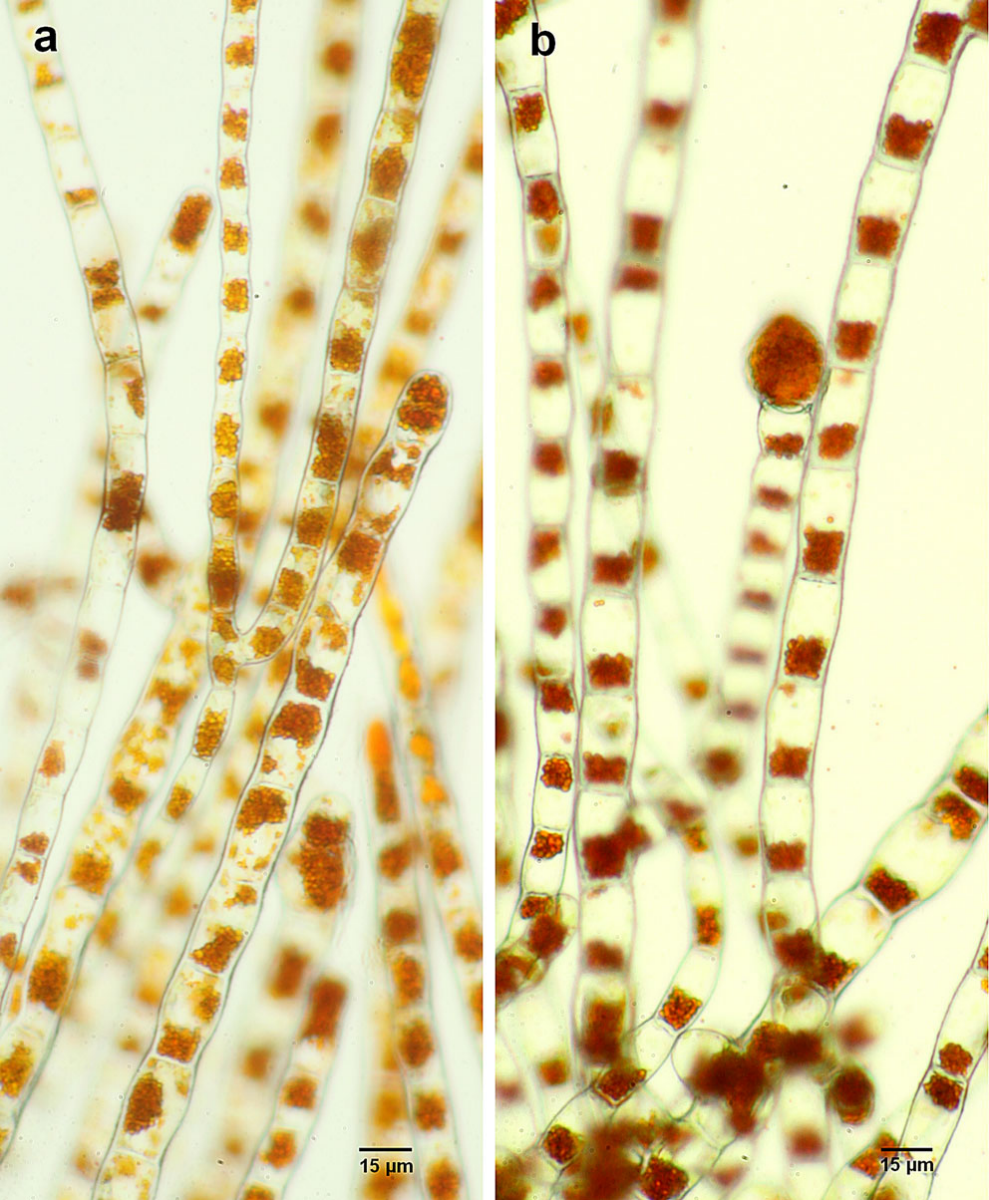
Isolated trentepohlialean photobiont from the lichen *Gyalecta jenensis* compared to the algal strain *Trentepohlia aurea* SAG483-1 (*free-living*) originated from the culture collection of Göttingen. **a** Algal filaments of photobiont *T. aurea* grown in liquid Woods Hole MBL medium; **b** Algal filaments of free-living *T. aurea* grown on solid Woods Hole MBL medium

Finally, we found that *Trentepohlia aurea* (Fig. 2a and b) is the typical photobiont of *Gyalecta jenensis* (clade Ta)*.* This has previously been suspected as free-living stages of this species are usually found adjacent to the thalli or growing out from the upper surface.

This study included different lichen genera and collection sites in temperate Europe and revealed that the species diversity of Trentepohliaceae in lichens from this region appears to be limited*.* Low photobiont variation was also shown previously for trentepohlialean lichens from Japan using culture experiments (Nakano 1988). On the other hand, it appears that at least some bark-inhabiting lichens may switch their trentepohlialean photobionts (Nelsen et al. 2011). This parallels the situation in lichens with coccoid green algae, where selectivity patterns have been found to vary in different lineages (Yahr et al. 2006). The classification of genera belonging to *Trentepohlia*, based on phenotypic characters, appears to be complicated by morphological polymorphism and it needs to be clarified as to whether widely distributed conspecific strains actually belong to the same lineage.

## Electronic supplementary material

Below is the link to the electronic supplementary material.

ESM 1 (DOCX 16 kb)

ESM 2 (DOCX 145 kb)

ESM 3 (DOCX 936 kb)
