# Supplementary material for: New insights into diversity and selectivity of trentepohlialean lichen photobionts from the extratropics
Source: Symbiosis. 2014 Jun 21;63(1):31–40. doi: 10.1007/s13199-014-0285-z (PMC4110408; doi:10.1007/s13199-014-0285-z)
Supplement: Supplementary file 1 — (DOCX 16 kb) [file 13199_2014_285_MOESM1_ESM.docx]

**Electronic Supplementary Material 1**

**New insights into diversity and selectivity of trentepohlialean lichen photobionts from the extratropics**

Journal: Symbiosis

Christina Hametner^1^, Elfriede Stocker-Wörgötter, and Martin Grube

^1^ Department of Organismic Biology, University of Salzburg, Hellbrunnerstraße 34, 5020 Salzburg, Austria, Tel. +43-662-80445528, Email. [Christina.Hametner2@sbg.ac.at](mailto:Christina.Hametner2@sbg.ac.at);

**Table S1** Strain numbers and descriptions of cultures used for primer design

| **Strain number** | **Description** |
| --- | --- |
| Tre 2 | photobiont of *Cystocoleus ebeneus* collected from Austria |
| TreFl 13 | photobiont of *Graphis scripta* collected from Austria |
| TreFl 14 | photobiont of *Graphis scripta* collected from Italy |
| TreFl 25 | photobiont of *Gyalecta jenensis* collected from Austria |
| TreFl 53 | photobiont of *Graphis submarginata* collected from Argentina |
| TreFl 55 | photobiont of *Graphis lineola* collected from Argentina |
| TreFl 56 | photobiont of *Phaeographis* sp. collected from Argentina |
| TreFl 57 | photobiont of *Graphis leptocarpa* collected from Argentina |
| TreFl 62 | photobiont of *Graphis propinqua* collected from Argentina |
| TreFl 69 | photobiont of *Graphis apertella* collected from Argentina |

**Table S2** Substitution models and settings of phylogenetic analyses with single ITS-alignments of diverse clades

| **clades** | **substitution model** | **jModelTest**  **parameters** | **generations** | **sample value** | **burnin** |
| --- | --- | --- | --- | --- | --- |
| Ce and Ld2 | SYM+I | yes | 2 000 000 | 1000 | 500 |
| Ta and Td | TrN+G | yes | 2 000 000 | 1000 | 500 |
| GPD | GTR | no | 2 000 000 | 1000 | 500 |
| Gs | F81 | no | 2 000 000 | 1000 | 500 |
| R and Ld1 | GTR+G+I | yes | 3 000 000 | 1000 | 750 |
