# Supplementary material for: New insights into diversity and selectivity of trentepohlialean lichen photobionts from the extratropics
Source: Symbiosis. 2014 Jun 21;63(1):31–40. doi: 10.1007/s13199-014-0285-z (PMC4110408; doi:10.1007/s13199-014-0285-z)
Supplement: Supplementary file 3 — (DOCX 936 kb) [file 13199_2014_285_MOESM3_ESM.docx]

**Electronic Supplementary Material 3**

**New insights into diversity and selectivity of trentepohlialean lichen photobionts from the extratropics**

Journal: Symbiosis

Christina Hametner^1^, Elfriede Stocker-Wörgötter, and Martin Grube

^1^ Department of Organismic Biology, University of Salzburg, Hellbrunnerstraße 34, 5020 Salzburg, Austria, Tel. +43-662-80445528, Email. [Christina.Hametner2@sbg.ac.at](mailto:Christina.Hametner2@sbg.ac.at);


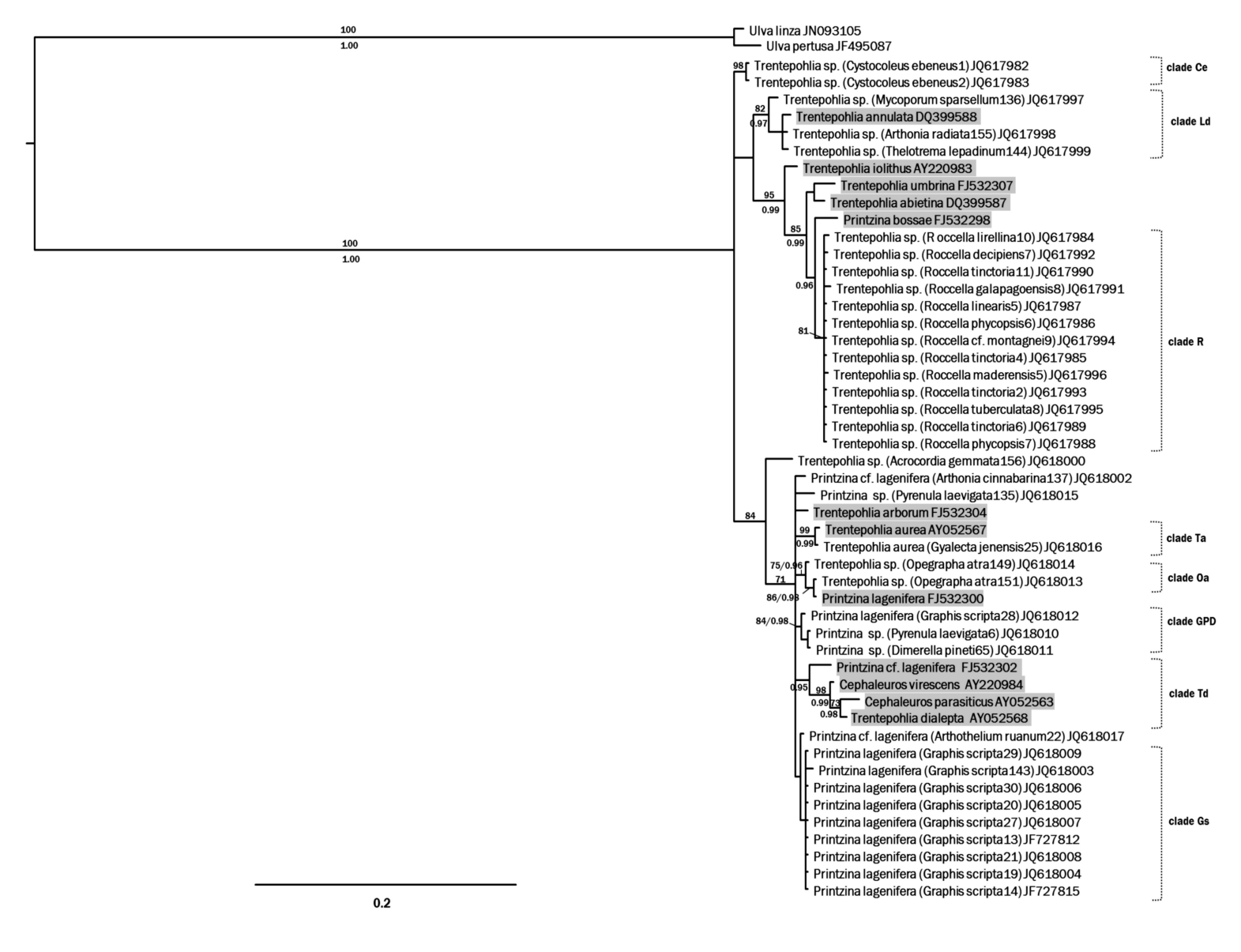


**Fig.4** Consensus tree of lichenized and free-living Trentepohliaceae based on 18S rRNA gene. Branches with bootstrap support (MPB) ≥ 70% and posterior probabilities (PP) ≥ 0.95 were considered as strongly supported. MPB values were illustrated above the branches, whereas PP values were shown below. The bar specifies the substitutions per site. The lichen specimen and specimen number of the photobiont strains is given in brackets. The trentepohlialean algae in free-living stage are grey-highlighted. The tree was rooted using two outgroup sequences of the genus *Ulva*


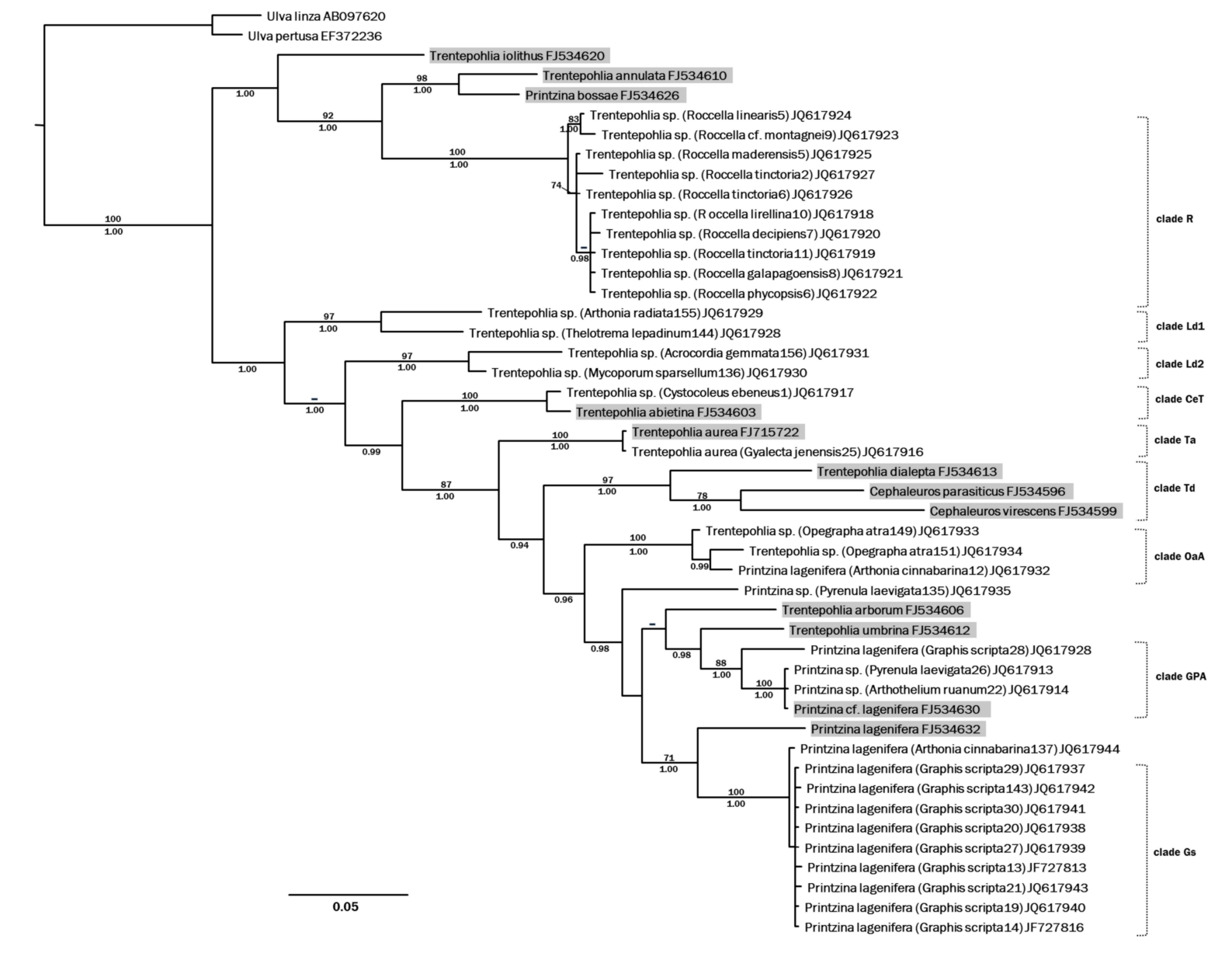


**Fig. 5** Consensus tree of lichenized and free-living Trentepohliaceae based on the *rbc*L gene. Branches with bootstrap support (MPB) ≥ 70% and posterior probabilities (PP) ≥ 0.95 were considered as strongly supported. MPB values were illustrated above the branches, whereas PP values were shown below. The bar specifies the substitutions per site. The lichen specimen and specimen number of the photobiont strains is given in brackets. The trentepohlialean algae in free-living stage are grey-highlighted. The tree was rooted using two outgroup sequences of the genus *Ulva*
